# Sleep Duration and Sun Exposure in Portuguese Children: Associations With Obesity Indicators

**DOI:** 10.1002/ajhb.70333

**Published:** 2026-09-20

**Authors:** Elizabete A. dos Santos, Aristides Machado‐Rodrigues, Daniela Rodrigues, Maria‐Raquel G. Silva, Helena Nogueira, Cristina Padez

**Affiliations:** ^1^ Research Centre for Anthropology and Health, Department of Life Sciences University of Coimbra Coimbra Portugal; ^2^ Department of Nutrition Paulista School of Medicine (EPM) of the Federal University of São Paulo (Unifesp) São Paulo Brazil; ^3^ Faculty of Sport Sciences and Physical Education University of Coimbra Coimbra Portugal; ^4^ FP‐I3ID, FP‐BHS, CEBIMED and Faculty of Health Sciences University Fernando Pessoa Porto Portugal; ^5^ Comprehensive Health Research Centre, Nova Medical School Nova University of Lisbon Lisbon Portugal; ^6^ Department of Geography and Tourism University of Coimbra Coimbra Portugal

**Keywords:** childhood obesity, children, pediatric public health, sleep duration, sun exposure

## Abstract

**Introduction:**

Sleep duration, sun exposure, and obesity are important and potentially interconnected factors in child health. In this study, we aimed to analyze the associations between sleep duration, sun exposure, and obesity indicators in Portuguese children.

**Methods:**

This cross‐sectional study included 4755 children aged 3–10 years from public and private schools across Portugal. Parents reported children's sleep duration, sun exposure, screen time, active play, and physical activity using validated questionnaires. Trained professionals measured weight and height to calculate Body Mass Index (BMI) and assessed skinfold thickness to estimate body fat percentage (%BF). Multiple linear regression analyses, stratified by sex and adjusted for demographic, behavioral, and anthropometric factors, analyzed associations between sun exposure and sleep duration.

**Results:**

In partially adjusted models (Model 1), negative associations persisted among boys, and positive associations emerged in girls for average sleep duration (*β* = 2.11; 95% CI: 0.25 to 3.96; *p* = 0.026) and weekdays (*β* = 2.40; 95% CI: 0.47 to 4.33; *p* = 0.015). In fully adjusted models (Model 2), the negative association remained only for boys on Sundays (*β* = −4.57; 95% CI: −8.22 to −0.91; *p* = 0.014), with no significant associations in girls.

**Conclusions:**

Sun exposure exhibits modest, sex‐specific associations with sleep duration in children. These findings emphasize the importance of considering behavioral and environmental factors when designing interventions to promote healthy sleep, support growth, and prevent childhood obesity.

## Graphical abstract

Overview of the study design and main findings on the associations among sun exposure, sleep duration, and obesity indicators in Portuguese children.

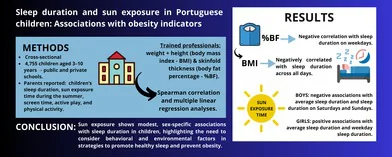

## 1 Introduction

Sleep is essential in promoting physical and mental health and supporting holistic development during childhood. Robust and consistent evidence demonstrates that adequate sleep duration is associated with lower body adiposity and better emotional outcomes, underscoring its importance for healthy growth (Matricciani et al. 2019). Conversely, insufficient sleep has been linked to adverse metabolic consequences, such as obesity, hypertension, and insulin resistance (Fobian et al. 2018; Kanellopoulou et al. 2021; Danial et al. 2023).

Sleep deprivation in children results from multiple factors, including biological determinants (such as age, puberty status, and nutritional profile), environmental exposures (e.g., heavy metals, air pollution, tobacco), interpersonal factors (e.g., family dynamics, screen use, diseases), behaviors, and sociocultural aspects (e.g., housing conditions, parental education, cultural norms) (Liu et al. 2024). Given this complexity, childhood sleep disturbances represent a significant public health concern that requires attention for effective prevention and management.

Sleep duration and timing are regulated by the interaction between two complementary processes: circadian rhythmicity (Process C) and sleep homeostasis (Process S). The circadian system, coordinated by the suprachiasmatic nucleus (SCN) of the hypothalamus, synchronizes sleep–wake rhythms with the environmental light–dark cycle, whereas homeostatic sleep pressure increases progressively during wakefulness and decreases during sleep (Reichert et al. 2016; Schwartz and Klerman 2019). Adenosine is considered an important mediator of this homeostatic process and contributes to the accumulation of sleep pressure during prolonged wakefulness (Reichert et al. 2016). In addition, hypothalamic neuronal systems contribute to sleep–wake regulation, particularly orexin/hypocretin neurons, which maintain arousal, and melanin‐concentrating hormone neurons, which are implicated in sleep regulation (Ono and Yamanaka 2017).

Hormone synthesis, secretion, and release are closely associated with circadian rhythms and sleep. At the same time, endocrine activity can, in turn, influence sleep duration and quality, demonstrating a bidirectional relationship between sleep and hormonal regulation (Beroukhim et al. 2022). The hypothalamic–pituitary–adrenal (HPA) axis is closely linked to the circadian system and contributes to endocrine regulation of the sleep–wake cycle. Environmental light signals reach the SCN, the central circadian pacemaker, which synchronizes melatonin secretion by the pineal gland and modulates cortisol secretion by regulating the HPA axis (Robertson‐Dixon et al. 2023; Androulakis 2021). These hormones have complementary circadian profiles: melatonin typically rises in the evening and peaks at night, signaling biological night, whereas cortisol reaches its highest concentrations in the early morning and progressively declines throughout the day (Robertson‐Dixon et al. 2023; Andreadi et al. 2025). Sleep and HPA‐axis activity are bidirectionally related, and disruptions in sleep or environmental light exposure may alter these hormonal rhythms and contribute to circadian misalignment (Androulakis 2021; Robertson‐Dixon et al. 2023).

Chronotype, defined as an individual preference for the timing of sleep and activity across the 24‐h day, is another important component of sleep–wake regulation. Chronotype varies throughout development and is influenced by biological and environmental factors, including age, sex, light exposure, and social schedules. Children generally show earlier circadian preferences, with a progressive shift toward eveningness beginning in childhood and becoming more pronounced during adolescence (Randler 2016; Silva et al. 2025). Evening chronotype has been associated with later sleep timing, greater social jet lag, poorer sleep patterns, and in some studies, higher adiposity and adverse behavioral outcomes in children and adolescents (Eid et al. 2020; Silva et al. 2025).

Sleep also varies seasonally and is influenced by demographic and geographic factors. Sleep patterns vary across the year, with longer sleep duration generally observed in winter than in summer (Scott et al. 2025). Seasonal variation in sleep duration also differs geographically, with sleep duration about 15–25 min longer in winter in the Northern Hemisphere and 15–20 min shorter in summer in the Southern Hemisphere. These variations decrease toward the equator (Scott et al. 2025). In children, seasonal and weekly variations have also been reported, with later sleep and wake times on weekends and earlier bedtimes and longer sleep duration during winter (Pal et al. 2023).

Sleep is part of a 24‐h cycle that coexists with other lifestyle behaviors, namely physical activity (Silva and Paiva 2019a) and sedentary time, which influence health (Matricciani et al. 2019). In this context, sun exposure time also plays an important role, not only in the sleep–wake cycle (Silva and Paiva 2019b), but also in cutaneous vitamin D3 synthesis. Moreover, sun exposure time is positively associated with time spent in active play and total physical activity in children (Dos Santos et al. 2023). From this integrated perspective, it becomes essential to consider sleep within the overall daily activity pattern (Silva et al. 2018) rather than in isolation (Matricciani et al. 2019).

Lifestyles increasingly confined to indoor environments, involving high screen time (Rodrigues et al. 2024), combined with heightened concerns about skin cancer, have significantly decreased sun exposure time (Lucas et al. 2013; Palacios and Gonzalez 2014; Alfredsson et al. 2020; Shahudin et al. 2020). Cutaneous vitamin D synthesis depends on several factors, including skin phototype. Human skin pigmentation represents a complex evolutionary adaptation to ultraviolet radiation (UVR), shaped by interactions among genetic, environmental, geographic, and cultural factors (Jablonski and Chaplin 2000, 2010; Jablonski 2021). Melanin modulates UVR penetration into the skin and therefore affects the biological consequences of solar exposure, including vitamin D synthesis. These relationships reflect an evolutionary balance between protection against excessive UVR exposure and maintenance of sufficient UVB penetration for vitamin D synthesis, with additional influences from latitude, diet, genetic variability, and cultural practices (Lucock et al. 2022; Lucock 2023). Time spent outdoors, clothing, sunscreen use, dietary intake, and genetic predisposition also affect vitamin D synthesis (Fleury et al. 2016; Kift et al. 2018). Additionally, cultural norms and social barriers may limit sun exposure and shape outdoor behavior patterns (Kift et al. 2018).

Like sleep disorders, vitamin D deficiency in children is a global public health issue (Ní Chaoimh et al. 2018; Mutua et al. 2020; Radonsky et al. 2024). Emerging evidence indicates insufficient vitamin D levels are associated with shorter sleep duration and lower sleep efficiency (Al‐Shawwa et al. 2020; Sung et al. 2021; Zhao et al. 2021). Both factors are also linked to excess weight and increased adiposity in childhood (Fiamenghi and Mello 2021; Pérez‐Bravo et al. 2022; Yuan et al. 2024). Despite these associations, few studies have examined the interplay between sleep duration, sun exposure, and obesity indicators in an integrated manner. Therefore, this study investigates whether sun exposure time is associated with sleep duration and whether sleep duration is associated with obesity indicators (body fat percentage and Body Mass Index [BMI]) in Portuguese children.

## 2 Materials and Methods

This study followed the Strengthening the Reporting of Observational Studies in Epidemiology (STROBE) guidelines (Von Elm et al. 2008).

### 2.1 Study Design

This observational, cross‐sectional study was conducted as part of the project “Inequalities in childhood obesity: The impact of the socio‐economic crisis in Portugal from 2009 to 2015.”

### 2.2 Participants

Children aged 3–10 years (50.1% female) were recruited from public and private schools (*n* = 118) located in the districts of Coimbra (*n* = 2980), Lisbon (*n* = 3066), and Porto (*n* = 2426). Data collection occurred between November 2016 and April 2017, resulting in a total sample of 8472 participants. Participation rates were 58% in Coimbra, 67% in Lisbon, and 60% in Porto. For the present analysis, only children whose guardians provided information on sun exposure time were included (*n* = 4755).

### 2.3 Ethical Aspects

The study was approved by the Research Ethics Committee of the National Data Protection Commission (CNPD; Ref. 745/2017) and received authorization from the Directorate‐General for Innovation and Curriculum Development (DGIDC), a body of the Ministry of Education. Written informed consent was obtained from parents or legal guardians.

### 2.4 Sociodemographic and Behavioral Characteristics

Data for the project were gathered through a parental survey and direct observations. Parents provided information regarding the child's age and sex, daily behaviors (sleep duration, sun exposure, screen time, active play, and physical activity), and the family's socioeconomic status (SES)/education level using a validated questionnaire. The survey was filled out at home and returned within 1 week, along with the signed consent form. This enabled the second data collection phase, which consisted of anthropometric measurements of the children. Further details on the variables are provided below.

#### 2.4.1 Sleep Duration

Parents reported their child's average sleep duration (in minutes) for weekdays (Monday–Friday), Saturdays, and Sundays. Sleep was considered adequate when children aged 3–5 years slept at least 600 min (10 h) per day and when children aged 6–12 years slept at least 540 min (9 h) per day, according to the National Health Service of the Portuguese Ministry of Health (National Health Service 2025).

#### 2.4.2 Sun Exposure Time

Sun exposure time was assessed using a questionnaire adapted from Hanwell et al. (2010). Although we collected data between November 2016 and April 2017, parents retrospectively reported their child's usual average daily sun exposure during the summer. Response options were: at least 15 min, about 1 h, about 2 h, or more than 2 h. The questionnaire also collected information on sunscreen use when the child left the house and on the body areas exposed to sunlight, including the face, hands, arms, and legs.

#### 2.4.3 Screen Time and Active Play

Parents also reported the time their children spent using multiple screen‐based devices (e.g., TV, computer, video games, smartphone, and tablet) and engaging in active play (i.e., outdoor physical activities) separately for weekdays and weekends. The daily duration was reported in categories: none, up to 1, 1, 2, 3, 4, and more than 4 h.

#### 2.4.4 Physical Activity

Questions about activities performed outside school assessed weekly physical activity (in total minutes). Parents indicated whether their child participated in organized sports outside school hours. If so, they separately reported the type of activity, weekly frequency (days per week), and total duration (minutes) for both weekdays and weekends.

#### 2.4.5 Socioeconomic Status (SES)

Since Portugal lacks an official measure of SES, the highest level of education attained by either parent was used as a proxy, following prior studies (Douglas‐Hall and Chau 2007; Rodrigues et al. 2021; Ware 2019). In single‐parent households, the education level of the child's primary guardian was used. Education level was categorized based on the Portuguese educational system as follows: Low—both parents with sub‐secondary education (≤ 9 years); Medium– at least one parent with secondary education (10–12 years); High—at least one parent with higher education (> 12 years; university degree).

### 2.5 Anthropometric Variables

During school visits, trained professionals measured weight, height, and skinfold thicknesses using a standardized protocol. Weight was measured using a portable digital scale with a maximum capacity of 150 kg and an accuracy of 0.1 kg. Height was measured with a wall‐mounted stadiometer, accurate to 0.1 cm. Each measurement was taken in duplicate to ensure reliability, and the mean value was used. Subsequently, Body Mass Index (BMI) was calculated using the equation proposed by Keys et al. (1972): BMI (kg/m^2^) = Weight (kg)/Height^2^ (m). Nutritional status was classified using the cutoff points defined by the International Obesity Task Force (IOTF) (Cole and Lobstein 2012).

Triceps and subscapular skinfolds were measured using a calibrated skinfold caliper (adipometer). The triceps skinfold was measured at the midpoint between the acromion and olecranon processes, with the skinfold (skin + subcutaneous fat) gently separated from the underlying muscle. The caliper was applied at a 90° angle while the child stood with the arm relaxed at their side. The subscapular skinfold was measured immediately below the inferior angle of the scapula. The skinfold was lifted 1 cm below this point, forming an approximate 45° angle between the skinfold and the spine. The child stood with arms and shoulders relaxed during the measurement. All skinfold measurements were taken in triplicate, and the mean was used for analysis.

Body fat percentage (%BF) was estimated using the predictive equations proposed by Slaughter et al. (1988).

$$\%\mathrm{BF}\;\text{Girls}=1.33\;\left(\Sigma \;\mathrm{TS}+\mathrm{SS}\right)-0.013\;{\left(\Sigma \;\mathrm{TS}+\mathrm{SS}\right)}^{2}–2.5$$

$$\%\mathrm{BF}\;\text{Boys}=1.21\;\left(\Sigma \;\mathrm{TS}+\mathrm{SS}\right)-0.008\;{\left(\Sigma \;\mathrm{TS}+\mathrm{SS}\right)}^{2}–1.7$$

### 2.6 Statistical Analyses

All categorical variables (sun exposure, active play, and screen time) were initially converted into continuous variables by assigning average values to the respective response categories. Weekly and weekend durations for screen time (aggregated across all electronic devices) and active play were summed to compute the total weekly time for each variable. Values initially expressed in hours were converted into minutes (e.g., 2.5 = 2 h + 0.5 × 60 = 150 min). Similarly, overall mean daily sleep duration was calculated by averaging mean weekday sleep duration and mean weekend sleep duration.

A descriptive analysis was conducted using measures of central tendency, with results expressed as means and standard deviations. The distribution of all variables was assessed using the Shapiro–Wilk test.

To compare differences in anthropometric measures and behavioral variables (i.e., time spent in active playtime, organized sports, screen‐media use, as well as sleep and sun exposure duration) by sex and age group (3–5 years/preschoolers vs. 6–10 years/school‐aged children), the Mann–Whitney‐Wilcoxon (Ranksum) and Kruskal‐Wallis tests were applied. Differences in sleep duration were also analyzed according to SES and sun exposure time.

Spearman correlation analyses assessed the association between sleep, sun exposure, and obesity indicators (BMI and %BF). Finally, multiple linear regression analyses, stratified by sex, were conducted using sun exposure as the independent variable and sleep duration (weekdays, Saturdays, Sundays, and overall mean daily sleep duration) as the dependent variable. First, a crude model was conducted. Then, model 1 used age and SES as adjustment variables. Model 2 included all variables from Model 1, with additional adjustment for BMI, %BF, and the time spent in active play, screen time, and organized sports.

STATA software version 13 (Stata Corp LP) was used to perform the analyses, and a significance level of 5% (*α* = 0.05) was considered for all statistical tests.

## 3 Results

A total of 4755 children with an average age of 7.11 ± 1.91 years were evaluated. Daily sun exposure time did not differ significantly by sex. However, girls generally demonstrated higher sleep duration on weekends (*p* < 0.0001), as well as higher BMI and body fat percentage compared to boys. The general characteristics of the sample according to age group and sex are presented in Table 1.

**TABLE 1:** General characterization of the participants (*n* = 4755), according to children's school‐age.

	Total (*n* = 4755)	Preschoolers (3–5) (*n* = 1, 399)		School‐aged children (6–10) (*n* = 3356)	
	Mean (SD)	Mean (SD)		Mean (SD)	
		Boys (*n* = 738)	Girls (*n* = 661)	Boys (*n* = 1633)	Girls (*n* = 1723)
Weight (kg)	25.55 (±7.66)	18.72 (±3.03)	18.71 (±3.35)	28.20 (±6.81)	28.58 (±7.48)**
BMI (kg/m^2^)	16.81 (±2.38)	16.17 (±1.39)	16.32 (±1.72)	16.93 (±2.47)	17.16 (±2.73)**
Body fat (%) *n* = 4680	18.30 (±6.20)	15.63 (±3.70)	17.71 (±4.20)	17.89 (±7.32)	20.04 (±5.99)**
Sun exposure time (min/day) *n* = 4755	210 (±45)	210.6 (±44.4)	205.8 (±46.2)	210.6 (±44.4)	210.6 (±45)*
Active play time (min/day) *n* = 4638	219 (±76.2)	240.6 (±)	223.2 (±81)	214.8 (±69.6)	211.8 (±76.8)**
Physical activity practice (min/day) *n* = 2698	147.91 (±107.53)	103.84 (±70.98)	105.26 (±68.34)	169.01 (±100.42)	153.84 (±125.76)**
Screen time (min/day) *n* = 4727	117.6 (±37.8)	112.8 (±36)	108 (±34.2)	124.8 (±37.8)	117 (±37.8)**
Sleep duration					
Weekdays (min/day)	603.44 (±35.74)	614.44 (± 36.08)	614.92 (± 36.95)	599.73 (± 33.27)	597.77 (± 35.35)**
*n* = 4664					
On Saturdays (min/day)	641.21 (±51.44)	643.80 (± 51.72)	650.23 (± 47.11)	632.06 (± 50.96)	645.33 (± 52.15)**
*n* = 4637					
On Sundays (min/day) *n* = 4613	638.35 (±49.47)	637.14 (± 50.49)	645.11 (± 47.20)	631.19 (± 48.80)	643.06 (± 49.66)*
Average (min/day) (*n* = 4683)	621.61 (± 35)	627.63 (± 36.84)	631.26 (± 34.50)	615.56 (± 35.53)	621.04 (± 34.52)**

*Note:* Mann–Whitney *U* test: *p < 0.05, **p < 0.0001.

Abbreviations: BMI, body mass index; SD, standard deviation.

As shown in Table 1, when stratifying data by age group and sex, all variables assessed—including sun exposure time, sleep duration, physical activity, active play, screen time, weight, BMI, and body fat percentage—showed statistically significant differences between preschoolers and school‐aged children. School‐aged children had higher sun exposure and screen time, and greater participation in organized physical activity, but lower sleep duration and active play compared to preschoolers. Within each age group, girls generally exhibited higher sleep duration and body fat percentage than boys. Notably, the average sleep duration in both age groups remained within the recommended values, indicating that most children achieved adequate sleep despite these differences.

Overall, 5.1% (*n* = 237) of the children had sleep duration below the recommended values. Among preschoolers, 15.4% (*n* = 213) slept less than 600 min per night, while only 0.7% (*n* = 24) of school‐age children slept less than 540 min per night. The average sleep duration was shorter on weekdays (603.44 min) compared to Saturdays (641.21 min) and Sundays (638.35 min).

**FIGURE 1:**
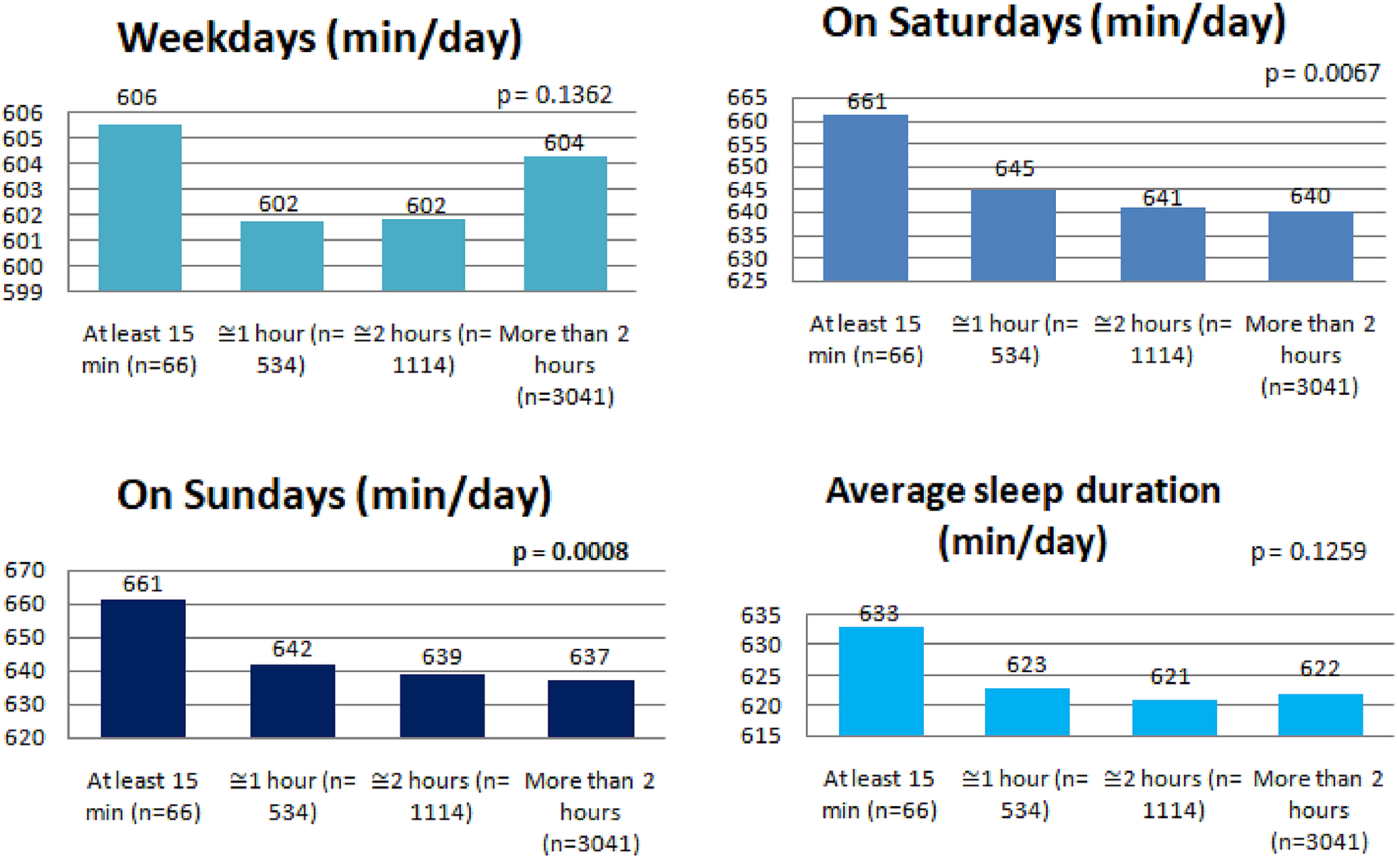
Association between weekend sun exposure and sleep duration in Portuguese children.

**FIGURE 2:**
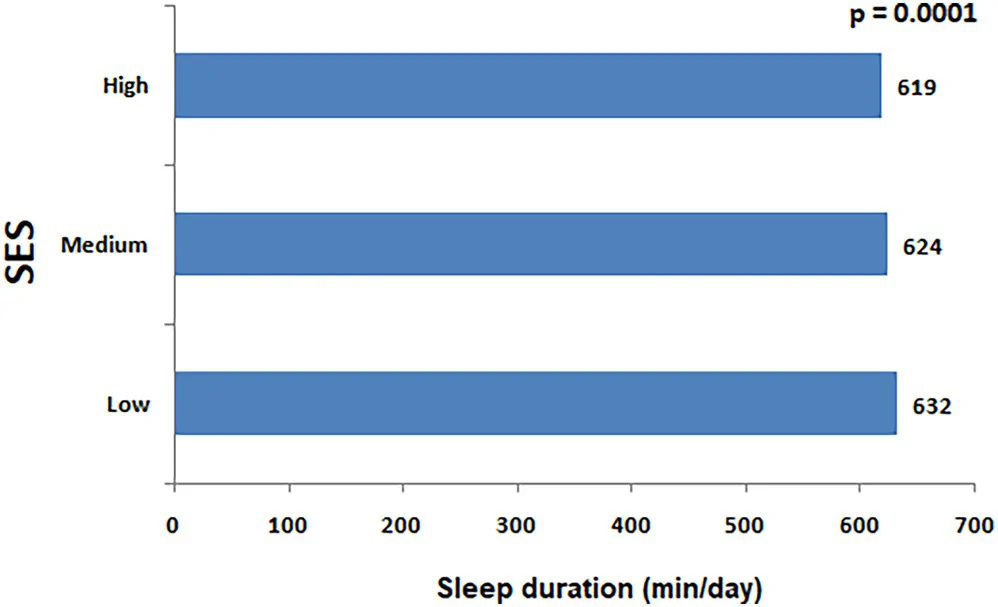
Average sleep duration according to socioeconomic status (SES).

Weekend sun exposure showed a negative association with sleep duration, with less exposure observed among children who slept more on weekend days (Saturdays, *p* = 0.0067 and Sundays, *p* = 0.0008) (Figure 1). Moreover, children in the lower SES group had a higher average sleep duration (632 min/day) than those in the higher SES group (619 min/day; *p* = 0.0001) (Figure 2).

Correlation analyses in the total sample showed a weak, positive association between weekly sleep duration and sun exposure time (*r* = 0.0334; *p* = 0.0225). In contrast, negative correlations were observed between sun exposure time and sleep time on Saturdays (*r* = −0.0305; *p* = 0.0375) and Sundays (*r* = −0.0363; *p* = 0.0138). Regarding obesity indicators, a negative correlation was found between %BF and sleep duration during the week (*r* = −0.0497; *p* = 0.0007). BMI showed negative correlations with weekly sleep duration (*r* = −0.0957; *p* < 0.001), Saturdays (*r* = −0.0403; *p* = 0.0061), Sundays (*r* = −0.0345; *p* = 0.0190), and average sleep duration (*r* = −0.0751; *p* < 0.0001). No significant correlations were found between sun exposure time and obesity indicators (%BF: *r* = −0.0272; *p* = 0.0624; BMI: *r* = −0.0116; *p* = 0.4258).

Linear regression analyses stratified by sex are presented in Tables 2 and 3. In the unadjusted model, among boys, sun exposure time was negatively associated with average sleep duration (*β* = −3.21; 95% CI: −5.14 to −1.28; *p* < 0.001), on Saturdays (*β* = −6.37; 95% CI: −9.21 to −3.53; *p* < 0.001), and Sundays (*β* = −6.25; 95% CI: −8.98 to −3.52; *p* < 0.001). No significant associations were observed among girls in this model.

**TABLE 2:** Multiple linear regression in girls—Crude model; Model 1; Model 2.

Crude model						
Independent variable	Dependent variables	*β*	SE	*p*	95% CI	Adjusted *R* ^2^
Sun exposure time	Sleep duration–Weekdays	1.91	1.011	0.059	−0.07 to 3.89	0.001
	Sleep duration–On Saturdays	0.99	1.412	0.484	−1.78 to 3.76	−0.000
	Sleep duration–On Sundays	−0.08	1.362	0.954	−2.75 to 2.59	−0.000
	Average sleep duration	1.43	0.959	0.135	−0.44 to 3.31	0.001
*Model 1*						
Sun exposure time *Adjustment variables: age, SES.	Sleep duration–Weekdays	2.40	0.986	**0.015**	0.47 to 4.33	0.058
	Sleep duration–On Saturdays	1.93	1.397	0.168	−0.81 to 4.67	0.028
	Sleep duration–On Sundays	0.65	1.354	0.630	−2.00 to 3.31	0.019
	Average sleep duration	2.11	0.947	**0.026**	0.25 to 3.96	0.033
*Model 2*						
Sun exposure time *Adjustment variables: age, SES, BMI, BF%, active play, screen time, physical activity time	Sleep duration–Weekdays	1.32	1.270	0.300	−1.18 to 3.81	0.095
	Sleep duration–On Saturdays	0.48	1.812	0.793	−3.08 to 4.03	0.019
	Sleep duration–On Sundays	−0.32	1.793	0.859	−3.84 to 3.20	0.015
	Average sleep duration	0.81	1.220	0.509	−1.59 to 3.20	0.053

*Note:* Bold values indicate statistically significant associations (*p* < 0.05).

Abbreviations: %BF, body fat percentage; BMI, body mass index; CI, confidence interval; SE, standard error; SES, socioeconomic status.

**TABLE 3:** Multiple linear regression in boys–Crude model; model 1; model 2.

Crude model						
Independent variable	Dependent variables	*β*	SE	*p*	95% CI	Adjusted *R* ^2^
Sun exposure time	Sleep duration–Weekdays	0.46	0.984	0.638	−1.47 to 2.39	−0.000
	Sleep duration–On Saturdays	−6.37	1.449	**0.000**	−9.21 to −3.53	0.008
	Sleep duration–On Sundays	−6.25	1.392	**0.000**	−8.98 to −3.52	0.008
	Average sleep duration	−3.21	0.984	**0.001**	−5.14 to −1.28	0.004
*Model 1*						
Sun exposure time *Adjustment variables: age, SES.	Sleep duration–Weekdays	0.96	0.968	0.320	−0.94 to 2.86	0.052
	Sleep duration–On Saturdays	−4.45	1.438	**0.002**	−7.27 to −1.63	0.048
	Sleep duration–On Sundays	−4.72	1.394	**0.001**	−7.45 to −1.99	0.032
	Average sleep duration	−2.13	0.969	**0.028**	−4.03 to −0.23	0.055
*Model 2*						
Sun exposure time *Adjustment variables: age, SES, BMI, BF%, active play, screen time, physical activity time	Sleep duration–Weekdays	0.54	1.257	0.669	−1.93 to 3.00	0.073
	Sleep duration–On Saturdays	−2.44	1.909	0.201	−6.18 to 1.30	0.030
	Sleep duration–On Sundays	−4.57	1.863	**0.014**	−8.22 to −0.91	0.016
	Average sleep duration	−1.64	1.271	0.197	−4.13 to 0.85	0.049

*Note:* Bold values indicate statistically significant associations (*p* < 0.05).

Abbreviations: %BF, body fat percentage; BMI, body mass index; CI, confidence interval; SE, standard error; SES, socioeconomic status.

In Model 1, a negative association between sun exposure time and average sleep duration was observed among boys (*β* = −2.13; 95% CI: −4.03 to −0.23; *p* = 0.028), as well as on Saturdays (*β* = −4.45; 95% CI: −7.27 to −1.99; *p* = 0.002) and Sundays (*β* = −4.72; 95% CI: −7.45 to −1.99; *p* = 0.001). Among girls, positive associations were found between sun exposure time and average sleep duration (*β* = 2.11; 95% CI: 0.25 to 3.96; *p* = 0.026) and during the week (*β* = 2.40; 95% CI: 0.47 to 4.33; *p* = 0.015). In Model 2, among boys, the negative association between sun exposure time and sleep duration remained only for Sundays (*β* = −4.57; 95% CI: −8.22 to −0.91; *p* = 0.014). For girls, no significant associations were found in this model.

Finally, when the analyses were stratified by age group (preschoolers versus school age), no significant associations were found in sleep duration, sun exposure time, and obesity indicators (data not presented). A summary of the main findings is presented in Table 4.

**TABLE 4:** Summary of the main findings on the associations among sleep duration, sun exposure, and obesity indicators.

Association	Main finding	Interpretation
Sun exposure × sleep duration − boys	Negative associations were observed in the crude and partially adjusted models. In the fully adjusted model, the association remained significant only for Sunday sleep duration.	The association between sun exposure and sleep duration in boys was attenuated after adjustment for behavioral and anthropometric factors.
Sun exposure × sleep duration − girls	Positive associations with overall mean daily and weekday sleep duration were observed in Model 1. No significant associations remained in the fully adjusted model.	Associations observed in girls did not persist after full adjustment.
Sleep duration × BMI	Higher BMI was associated with shorter sleep duration on weekdays, Saturdays, Sundays, and overall mean daily sleep duration.	Higher BMI was consistently associated with shorter sleep duration.
Sleep duration × body fat percentage (%BF)	Higher %BF was associated with shorter weekday sleep duration.	Higher adiposity was associated with shorter sleep duration during weekdays.
Sun exposure × obesity indicators	No significant correlations were observed between sun exposure and BMI or %BF.	No evidence of an association between reported sun exposure time and obesity indicators was observed.
Age‐stratified analyses	No significant associations among sleep duration, sun exposure, and obesity indicators were observed after stratification by age group.	No evidence of age‐specific associations was observed.

## 4 Discussion

A positive correlation was observed between weekly sleep duration and sun exposure time. These results partially confirmed our hypothesis. However, this relationship was negative on Saturdays and Sundays, indicating that children who slept longer tended to have lower sun exposure during weekends. Additionally, %BF showed a negative correlation with sleep duration on weekdays, and BMI was negatively correlated with sleep duration across all days. In the sex‐stratified analysis, the adjusted model (for age and SES) showed a positive association between sun exposure and average and weekday sleep duration, but only among girls. Conversely, a negative association was observed for boys in both the crude and adjusted models (Model 1), which remained significant over the weekend. In Model 2, the negative association persisted only on Sundays.

The positive association between sleep duration and sun exposure on weekdays may be related to greater routine regularity, especially regarding bedtimes and wake‐up times, and exposure to outdoor environments during commuting to school or school‐based activities. In a study by Baradaran Mahdavi et al. (2020), which included 14 000 Iranian children and adolescents aged 7–18 years (CASPIAN‐V Study), sleep duration was positively associated with sunlight exposure, sex, age, BMI, and physical activity level, and negatively associated with coffee and tea consumption. The authors highlighted that greater exposure to sunlight, especially on the face, hands, arms, and feet, along with higher physical activity and better mental health, significantly reduced the likelihood of delayed sleep onset (after midnight). Increased sun exposure was linked to better sleep duration and earlier sleep onset, suggesting a potential role of sunlight on circadian rhythm in this age group.

Several studies reinforce the role of sunlight in regulating the circadian cycle (Wright Jr. et al. 2013; Stothard et al. 2017; Wams et al. 2017; Silva and Paiva 2019b). Two main effects of light are widely discussed in the sleep literature: the acute suppression of melatonin and the ability of light to shift the circadian phase (Blume et al. 2019; Silva and Paiva 2019b). Natural light has a broad spectrum that varies depending on geographic location and season, making it a critical biological synchronizer (Silva et al. 2016). On the other hand, although artificial light may appear similar to natural light to the human eye, it has distinct spectral characteristics depending on the source (e.g., incandescent, fluorescent, LED), which may have varying effects on the biological clock (Blume et al. 2019). Notably, blue light emitted by electronic devices such as smartphones and computers has been associated with circadian phase delays and later sleep onset (Wright Jr. et al. 2013). This context highlights the importance of monitoring screen time in children and adolescents, as numerous studies demonstrate associations between increased screen exposure and poorer sleep quality (Chaput et al. 2014; Hale and Guan 2015; Carter et al. 2016; Saunders et al. 2022).

The negative correlation between sleep duration and sun exposure on weekends suggests that extended sleep may reduce the time available for outdoor activities during peak sunlight hours. This behavior may reflect a tendency for “sleep compensation” on weekends. A study by Choi et al. (2020) involving Korean adults and older adults (aged 19–97) investigated the relationship between vitamin D status, sleep duration, and sun exposure. Although no significant differences in vitamin D levels or sleep duration were found among participants with adequate sun exposure, those with insufficient sun exposure and prolonged sleep showed significantly lower 25(OH)D levels, even after adjusting for confounding variables (lifestyle, age, sex, sociodemographic factors, presence of chronic diseases, among others). Despite being conducted in an older population, the study underscores the importance of examining the interplay between sun exposure, sleep, and vitamin D status. Similarly, a school‐based cross‐sectional study involving children and adolescents aged 8–14 years identified a significant positive correlation between 25(OH)D levels and sleep duration (*r* = 0.11; *p* < 0.05). In multivariate logistic regression analyses, vitamin D insufficiency or deficiency (25(OH)D ≤ 20 ng/mL) was associated with increased odds of short sleep duration (AOR = 1.67; 95% CI = 1.14–2.43). The authors also noted that 32.8% of the children assessed exhibited insufficient sleep (< 9 h per day) (Gong et al. 2018).

Although this study did not include 25(OH)D measurements, vitamin D deficiency in children has been associated with low sun exposure and reduced outdoor activity (Voortman et al. 2015; Oliosa et al. 2023). Given vitamin D's essential role in healthy growth and development during childhood (Yakoob and Lo 2017), promoting outdoor activity may support healthy lifestyle patterns in children. However, sun exposure time should not be treated as a direct proxy for vitamin D synthesis, as effective UVB exposure varies with factors such as season, latitude, time of day, skin phototype, and ambient UVB irradiance (Jablonski and Chaplin 2010; Lucock 2023).

The sex‐based differences observed in this study are also noteworthy. While girls showed a positive association between sun exposure and sleep duration on weekdays, boys demonstrated a negative association, particularly on weekends. This finding may be related to behavioral differences in childhood, such as increased screen time among boys, especially during weekends (Abdel Magid et al. 2021; Nagata et al. 2022).

Among Brazilian children and adolescents aged 7–13, factors such as age, morning classes, and full‐time school attendance were associated with shorter sleep duration during the week and longer on weekends. Moreover, girls tended to sleep more on weekends (Fumo‐Dos‐Santos et al. 2019). Our study also found that girls slept more, which is consistent with previous findings (Natal et al. 2009; Biggs et al. 2013; Baradaran Mahdavi et al. 2020).

The absence of significant associations in age‐stratified analyses may indicate that the relationship between sleep and sun exposure is more influenced by behavioral and environmental factors than by age per se. However, it is important to consider that children's routines can vary substantially with age, including parental supervision, screen time, and physical activity patterns—all of which may have attenuated or masked potential associations.

In addition to environmental and behavioral aspects, biological characteristics may also influence children's sleep and sun exposure patterns. This study included BMI and %BF as covariates in the regression models based on evidence linking excess weight to poorer sleep quality or shorter sleep duration during childhood (Miller et al. 2021; Morrissey et al. 2020). Furthermore, children with higher adiposity tend to engage in less outdoor physical activity (Cleland et al. 2008; Stone and Faulkner 2014; Zhang et al. 2020), which may limit their sunlight exposure and may influence circadian regulation.

Our analyses suggest that children who sleep longer tend to have lower %BF and BMI, while those who sleep less show higher values for these indicators, which is in line with previous studies (Morrissey et al. 2020; Xiu et al. 2020; Kanellopoulou et al. 2021; Chen et al. 2022). In a prospective study involving Swedish children aged 1–8 years, Danial et al. (2023) found that each hour of reduced sleep was associated with a 0.09 unit increase in BMI z‐score, suggesting that shorter sleep duration may contribute to an increased risk of overweight and obesity. It is important to note that, beyond sleep duration, other dimensions, such as sleep quality, efficiency, and bedtime/wake‐up time, may also influence body composition and should be considered in future investigations (Morrissey et al. 2020).

Although we did not find significant correlations between sun exposure time and adiposity indicators, previous evidence has shown that sun exposure has been associated with BMI, %BF, and skinfold thickness (Dos Santos et al. 2023). Moreover, several studies have demonstrated an inverse association between adiposity and serum 25(OH)D concentrations, indicating that higher levels of body fat may be related to lower availability of vitamin D in the body (Greene‐Finestone et al. 2017; Guo et al. 2021; Pérez‐Bravo et al. 2022).

Another interesting finding was the association between low SES and higher sleep duration. A cross‐sectional study of 6–9‐year‐olds exploring parenting practices and SES found that lower‐SES parents were more consistent in setting screen‐time rules, particularly regarding gaming. This finding suggests that parental rule‐setting may contribute to lower children's screen time, even when overall device access is limited (De Lepeleere et al. 2018). However, our study did not collect information on the context of screen use (e.g., alone or accompanied) or household rules regarding this behavior. Moreover, the literature presents conflicting findings: studies in different socioeconomic and cultural contexts have reported longer sleep durations among children from higher‐income and better‐educated families (Salway et al. 2019; Lee et al. 2022). These discrepancies indicate that the relationship between SES and sleep duration is complex and likely mediated by factors such as age, access to technology, daily activity load, family culture, and parenting practices (Cameron et al. 2022; Etindele Sosso et al. 2022; Hansen et al. 2023).

Among the main strengths of this study are the large sample size, which provided robust analytical power, and the inclusion of sun exposure time as a variable that remains underexplored in the literature. The detailed evaluation of sleep patterns on weekdays and weekends allowed for a more comprehensive understanding of children's routines. At the same time, stratified analyses by sex and age group revealed important behavioral differences. Combining BMI and %BF enriched the sample's nutritional characterization. Additionally, the statistical models were adjusted for relevant behavioral variables, including screen time, physical activity, active play, and SES, strengthening the interpretation of the observed associations and allowing for a more contextualized analysis.

This study presents some limitations that should be considered when interpreting the findings. First, we did not assess sun exposure separately for weekdays and weekends. In addition, although data collection occurred between November 2016 and April 2017, parents retrospectively reported their child's usual summer sun exposure. Thus, sun exposure and sleep duration did not necessarily cover the same period, and retrospective reporting may have increased recall bias. Although we collected information on sunscreen use and body areas exposed to sunlight, self‐reported sun exposure time should not be interpreted as a direct measure of effective UVB exposure or cutaneous vitamin D synthesis, which also depend on factors such as season, latitude, time of day, skin phototype, and ambient UVB irradiance. In particular, this study did not assess skin phototype. Serum 25(OH)D levels were also not measured, which prevented direct assessment of vitamin D status and its relationship with sun exposure and sleep duration. Furthermore, sleep duration was parent‐reported rather than objectively measured, and the study did not assess other relevant dimensions of sleep, including sleep timing, quality, efficiency, and chronotype. Finally, the cross‐sectional design limits the ability to establish temporal or causal relationships.

## 5 Conclusion

The main findings of this study indicate that sleep duration in Portuguese children is associated with environmental and behavioral factors, particularly sun exposure, with distinct patterns observed between boys and girls. Higher BMI and body fat percentage were also associated with shorter sleep duration. These findings help explain the complex interplay among sleep, sun exposure, adiposity, and other childhood lifestyle factors.

The observed sex‐specific patterns highlight the importance of considering individual and behavioral differences when investigating sleep and sun exposure. Given the cross‐sectional design and the limitations of self‐reported sun exposure, future longitudinal studies should incorporate objective measures of sleep, sun exposure, UVB exposure, and vitamin D status to clarify the direction and mechanisms underlying these associations. Moreover, more straightforward guidelines on appropriate sleep and sun exposure are needed, considering other relevant behaviors such as screen time. Effective interventions should address not only sleep quantity but also its timing and the quality and timing of sunlight exposure, particularly on weekends.

## Author Contributions

**Elizabete A. dos Santos:** writing – original draft, investigation, data curation, formal analysis. **Aristides Machado‐Rodrigues:** conceptualization, methodology, investigation, writing – review and editing. **Daniela Rodrigues:** writing – review and editing. **Maria‐Raquel G. Silva:** conceptualization, methodology, investigation, writing – review and editing. **Helena Nogueira:** conceptualization, methodology, investigation, writing – review and editing. **Cristina Padez:** conceptualization, methodology, investigation, funding acquisition, project administration, supervision, writing – review and editing.

## Funding

This study was financially supported by the Foundation for Science and Technology (Portugal) through grant PTDC/DTP‐SAP/1520/2014, with funds from COMPETE 2020, Portugal 2020, FEDER, and FCT. The funders had no role in the design, data collection, analysis, decision to publish, or preparation of the manuscript.

## Disclosure

The authors used ChatGPT (OpenAI) solely to assist with English‐language editing and grammatical review. The tool was not used for data collection, data analysis, statistical procedures, interpretation of the findings, or generation of scientific conclusions. All AI‐assisted revisions were critically reviewed and verified by the authors to ensure their accuracy, integrity, and consistency with the original scientific content. The authors take full responsibility for the final content of the manuscript.

## Ethics Statement

The study was approved by the Research Ethics Committee of the National Data Protection Commission (CNPD; Ref. 745/2017) and received authorization from the Directorate‐General for Innovation and Curriculum Development (DGIDC), a body of the Portuguese Ministry of Education. Written informed consent was obtained from the parents or legal guardians of all participating children.

## Conflicts of Interest

The authors declare no conflicts of interest.

## Data Availability

The data that support the findings of this study are available on request from the corresponding author. The data are not publicly available due to privacy or ethical restrictions.
